# Integrative meta-analysis identifies microRNA-regulated networks in infantile hemangioma

**DOI:** 10.1186/s12881-015-0262-2

**Published:** 2016-01-15

**Authors:** Natália Bertoni, Lied M. S. Pereira, Fábio E. Severino, Regina Moura, Winston B. Yoshida, Patricia P. Reis

**Affiliations:** Department of Surgery and Orthopedics, Faculty of Medicine, São Paulo State University-UNESP, Av. Prof. Montenegro, 18618-970 Botucatu, São Paulo Brazil

**Keywords:** Infantile hemangioma, MicroRNAs, Gene expression, Protein-protein interaction networks, Molecular pathogenesis, Treatment

## Abstract

**Background:**

Hemangioma is a common benign tumor in the childhood; however our knowledge about the molecular mechanisms of hemangioma development and progression are still limited. Currently, microRNAs (miRNAs) have been shown as gene expression regulators with an important role in disease pathogenesis. Our goals were to identify miRNA-mRNA expression networks associated with infantile hemangioma.

**Methods:**

We performed a meta-analysis of previously published gene expression datasets including 98 hemangioma samples. Deregulated genes were further used to identify microRNAs as potential regulators of gene expression in infantile hemangioma. Data were integrated using bioinformatics methods, and genes were mapped in proteins, which were then used to construct protein-protein interaction networks.

**Results:**

Deregulated genes play roles in cell growth and differentiation, cell signaling, angiogenesis and vasculogenesis. Regulatory networks identified included microRNAs miR-9, miR-939 and let-7 family; these microRNAs showed the most number of interactions with deregulated genes in infantile hemangioma, suggesting that they may have an important role in the molecular mechanisms of disease. Additionally, results were used to identify drug-gene interactions and druggable gene categories using Drug-Gene Interaction Database. We show that microRNAs and microRNA-target genes may be useful biomarkers for the development of novel therapeutic strategies for patients with infantile hemangioma.

**Conclusions:**

microRNA-regulated pathways may play a role in infantile hemangioma development and progression and may be potentially useful for future development of novel therapeutic strategies for patients with infantile hemangioma.

## Background

Infantile Hemangioma, a common benign tumor in childhood, occurs in 10 % of children, more frequently in prematures and females [1]. It shows a cycle with three phases: an initial proliferative phase (rapid growth during the first year), a plateau and an involution phase (spontaneous regression over 1–8 years) [2, 3]. In a study by Chang et al. [4], growth characteristics were examined in a large number (n = 526) of infantile hemangiomas and the results showed that infantile hemangioma growth occurred mainly in infancy, at a mean age of 3 months. Infantile hemangiomas may be deep or superficial, classified based on the depth of lesions. Deep infantile hemangiomas usually appear later in life and may be associated with a longer growth phase compared to the superficial form. Superficial infantile hemangiomas may be focal or segmental [4]. Segmental lesions are associated with a longer proliferative phase and could require a longer period of treatment [5].

The body area more frequently affected by hemangioma is the head and neck, mainly the face, with an association with the embryological development of the face [6]. Available treatment for patients with infantile hemangioma includes the use of corticosteroids and/or surgical resection of the tumor. The standard of care treatment strategy is the use of propranolol hydrochloride, a β-blocker entered as a safer form of treatment for proliferating infantile hemangioma [7, 8]. Although the advances in therapeutic strategies for infantile hemangioma, the main clinical problems are still the lack of reliable parameters able to distinguish proliferative from involuting IH lesions and the diverse response rates of patients to treatment.

Therefore, the identification of genetic and epigenetic alterations in proliferating and involuting infantile hemangioma lesions will likely contribute to better understand the underlying molecular mechanisms of development and progression of this disease, which is a leading cause of morbidity in affected children. Indeed, differences in the expression of genomic biomarkers have been reported in infantile hemangioma; e.g., insulin-like growth factor 2 (IGF-2) was found as highly expressed in proliferative lesions compared to involuting lesions [9].

In infantile hemangioma, neural crest markers (NG2 and nestin), pericytes markers (δ-like kinase, smooth muscle actin, calponin and CD90) and stem cell markers (OCT4, NANOG and SOX2) are frequently over-expressed both at mRNA and protein levels. In addition, pericytes (perivascular cells surrounding microvessels and that are related to the development and regulation of angiogenesis) and the derm of the face are derived from neural crest, suggesting that the neural crest may be involved in disease pathogenesis [10]. Importantly, expression of lymphatic endothelial hyaluronan receptor-1 (LYVE-1) has been reported in kaposiform hemangioendothelioma and tufted angioma [11]. LYVE-1 was detected as strongly expressed in proliferative infantile hemangiomas but not in pyogenic granulomas or intramuscular hemangioma lesions, suggesting an important role of these markers in the biology of infantile hemangioma [12]. microRNAs (miRNAs) play an important role in gene expression regulation and have been demonstrated to play a role in the pathogenesis of several human diseases [13]. miRNAs are small, non-coding RNAs containing ~18–24 nucleotides. They can bind to the 3’ and 5’ends of the mRNA, leading, in most cases, to translation inhibition or mRNA degradation [14, 15]. Furthermore, miRNAs are related to important biological processes, such as embryonic development, differentiation, apoptosis, cell proliferation [16–18] and oncogenesis [19–21]. To date, 2588 miRNAs were identified and characterized as to their sequence and function in the human genome (http://www.mirbase.org/cgi-bin/browse.pl?org=hsa) [22–26].

Different mechanisms can lead to deregulated miRNA expression, including genomic alterations, such as DNA gains or amplifications and mutations, epigenomic changes including DNA methylation and defects in miRNA biogenesis, including transcription and processing of miRNAs [27, 28]. miRNAs that are altered by these mechanisms may lead to deregulated gene expression.

The understanding of genetic and epigenetic mechanisms, such as deregulated miRNA and target gene expression, as well as molecular pathways regulated by miRNAs, may contribute for the development of new strategies for diagnosis and treatment of complex human diseases [13, 14, 16]. Currently, it is known that miRNAs and miRNA-target genes may represent useful biomarkers to help improve diagnosis, prognosis and treatment of human diseases, such as cancer [29, 30]. Although some gene expression studies have been previously published [31–37], there are no current data on miRNAs or deregulated protein-protein interaction networks in hemangioma. Such data integration strategy is important to understand the functional significance of deregulated genes, miRNAs and molecular pathways involved in hemangioma development and progression. In addition, miRNAs and their target genes may be clinically applicable as therapeutic targets. Indeed, a systematic integration of data derived from multiple sources may achieve the appropriate statistical power and lead to robust, reproducible and accurate predictions [38].

To the best of our knowledge, there are no studies on global miRNA expression in infantile hemangioma. A recent PubMed search (August 19, 2015) showed only one published study on the involvement of miRNAs in senile hemangioma [39], which reported decreased miR-424 expression and increased levels of CCNE1 and MEK1 proteins, which are targeted by miR-424, in patient samples. This study suggested that abnormal proliferation in senile hemangioma may be regulated, at least in part, by miR-424 [39].

Herein, we performed a comprehensive meta-analysis of gene expression data in infantile hemangioma and identified miRNAs as potential regulators of target genes in these tumors. Gene expression datasets were integrated with miRNAs for the identification of molecular pathways potentially involved in infantile hemangioma development and progression. These data may be clinically valuable to predict which infantile hemangioma lesions may respond and which lesions will be resistant to currently available treatment modalities. Furthermore, these data are useful for the identification of robust biomarkers applicable in the development of novel and better molecularly-targeted treatment strategies in infantile hemangioma.

## Methods

### Meta-analysis of gene expression data in infantile hemangioma

**Fig. 1:**
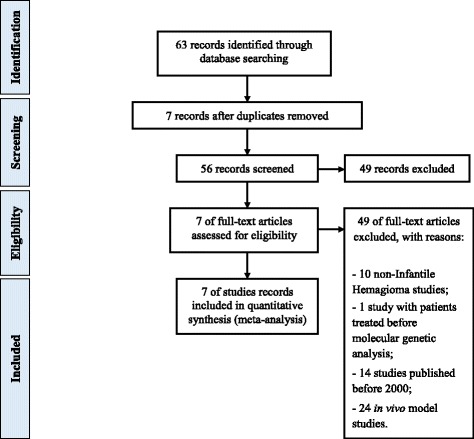
Flowchart of meta-analysis process

Meta-analysis study design followed the stages of the PRISMA Statement [40] (Fig. 1). Herein, we performed a meta-analysis of previously published gene expression data in infantile hemangioma, by searching PubMed (http://www.ncbi.nlm.nih.gov/pubmed). Key words used were: *“infantile hemangioma* AND *global gene expression”*, *“infantile hemangioma* AND *gene signature”*, *“infantile hemangioma* AND *microRNAs”*, *“microRNA in infantile hemangioma”*, *“infantile hemangioma* AND *microarray”*, *infantile hemangioma* AND *mRNA expression”*. Meta-analysis searches comprised studies published between the years of 2000–2015. Considering that our searches did not retrieve any records on miRNA studies in infantile hemangioma, we included only gene expression studies in this meta-analysis. Deregulated genes reported in selected studies were further used for bioinformatics prediction of miRNAs as potential regulators of gene expression, as described below.

Inclusion criteria were: gene expression data in primary patient samples of infantile hemangioma or pure cell populations of infantile hemangioma, any subtype of disease, inclusion of normal tissues for comparison, data subjected to independent validation. Exclusion criteria were: non- infantile hemangioma, patients treated before molecular genetic analysis and in vivo model studies.

### Identification of miRNAs as potential modulators of deregulated genes in infantile hemangioma

Deregulated genes identified in the meta-analysis were used for bioinformatics prediction of miRNAs as regulators of gene expression. We used microRNA Data Integration Portal, mirDIP [41], a computational tool that integrates several predicted and validated miRNA databases. mirDIP allows searching for genes that are targeted by miRNAs as well as for miRNAs predicted to regulate genes. Additionally, relevant biological pathways for differentially expressed genes were identified using Biological Networks Gene Ontology (BiNGO) tool, application available in Cytoscape v3.1.1 [42]. BiNGO allows recognizing which of Gene Ontology (GO) categories are statistically more represented in a specific set of genes. Protein-protein interaction (PPI) networks were then generated using Metasearch STRING v9.1 [43, 44] and visualization and annotation data of PPI and miRNA-gene interaction networks were generated using Cytoscape v3.1.1 [45, 46]. Furthermore, we identified drug-gene interactions using Drug-Gene Interaction Database (DGIdb), a database and web-interface for identifying known and potential drug-gene relationships. Genes were defined by Entrez Gene and Ensembl and matched with genes from drug-gene interactions and druggable gene categories. Drugs were defined by searching PubChem and then matched with drugs from drug-gene interaction data. Drug-gene interactions were obtained from multiple sources, including DrugBank, Therapeutic Target Database (TTD) and Pharmacogenomics Knowledge Base (PharmGKB) [47].

## Results

### Protein-protein interaction networks Identified in infantile hemangioma

According to the meta-analysis study design and the inclusion and exclusion criteria (Fig. 1), we selected 7 studies reporting gene expression data in infantile hemangioma [31–37] (Table 1). Altogether, these studies reported a total of 54 differentially expressed genes (36 over- and 18 under-expressed) in 98 patient samples (Table 2).

**Table 1:** Description of publicly available studies used in the meta-analysis

Reference ID	Sample size	Gene expression analysis and validation analysis platforms
[31]	6 hemangiomas and 7 normal term placental tissues	U95Av2 GeneChip oligonucleotide microarrays (Affymetrix)
[32]	7 hemangiomas (3 proliferating, 4 involuting) and 3 normal term placental tissues	Human Genome U133 Plus 2.0 (Affymetrix)
[33]	4 pairs of early proliferative stage and spontaneously early involution stage of the same hemangiomas, 11 hemangiomas (6 proliferative and 5 involuting), 5 controls (normal skin) Serum from 69 patients with hemangioma (46 proliferative and 23 involuting), 20 patients with venous malformations and 31 negative controls (children with cheilopalatognathus)	Illumina Human-6 bead chip, QRT-PCR
[34] GSE43742	HEMECs, HDMVECs, 16 infantile hemangioma, 4 normal controls (neonatal foreskin)	Illumina HumanHT-12 V4.0 expression beadchip Immunohistochemistry
[35]	hemSCs, bm-MPCs, HDMECs, cbEPCs and abEPCs	RQ-PCR, Functional assays, Immunofluorescence
[36]	HemSCs, HemECs, HDMECs and MSCs	RQ-PCR, Immunofluorescence
[37]	48 hemangiomas, 9 vascular malformations and vascular tumor specimens, 11 neonatal foreskin controls and HemECs from proliferating hemangioma	GeneFilter GF211 (Invitrogen)

*QRT-PCR* quantitative reverse-transcription polymerase chain reaction, *HEMECs* infantile hemangioma endothelial cells, *HDMVECs* dermal microvascular endothelial cells, *HemECs* hemangioma-derived endothelial cells, *hemSCs* proliferating hemangioma-derived CD133+ cells, *HDMECs* human dermal microvascular endothelial cells, *cbEPCs* cord blood endothelial progenitor cells, *abEPCs* adult blood endothelial progenitor cells, *bm-MPCs* bone marrow-mesenchymal progenitor cells, *HemSCs* hemangioma-derived stem cells, *MSCs* mesenchymal stem cells

**Table 2:** List of 54 deregulated genes identified in infantile hemangioma, as reported by the seven studies included in the meta-analysis

Gene symbol	Gene name	Gene function	Gene ID
Over-expressed			
*SMARCE1*	SWI/SNF related, matrix associated, actin dependent regulator of chromatin, subfamily e, member 1	chromatin remodelling	6605
*RGS5*	Regulator of G-protein signaling 5	cell signaling	8490
*CTAG2*	Cancer/testis antigen 2	autoimmunogenic tumor antigen	30848
*LTBP2*	Latent transforming growth factor beta binding protein 2	cell growth and differentiation	4053
*ANG*	Angiogenin, ribonuclease, RNase A family, 5	cell growth and differentiation	283
*IGF2*	Insulin-like growth factor 2	cell growth and differentiation	3481
*TBX2*	T-box 2	transcription factor	6909
*NOTCH3*	Notch 3	cell fate and signalling	4854
*HSD17B2*	Hydroxysteroid (17-beta) dehydrogenase 2	uncharacterized	3294
*TFPI2*	Tissue factor pathway inhibitor 2	tumor supressor	7980
*GNG11*	Guanine nucleotide binding protein (G protein), gamma 11	cell signaling	2791
*NID1*	Nidogen 1	cell interactions	4811
*COL4A1*	Collagen, type IV, alpha 1	basement membrane/metabolism	1282
*KDR*	Kinase insert domain receptor (a type III receptor tyrosine kinase)	cell growth and differentiation	3791
*FCGR2B*	Fc fragment of IgG, low affinity IIb, receptor (CD32)	immunocomplex phagocytosis/antibody production regulation	2213
*PLAGL1*	Pleiomorphic adenoma gene-like 1	tumor supressor	5325
*DLK1*	Delta-like 1 homolog (Drosophila)	cell growth and differentiation	8788
*JAM3*	Junctional adhesion molecule 3	cell adhesion	83700
*NID2*	Nidogen 2 (osteonidogen)	cell adhesion	22795
*MEOX2*	Mesenchyme homeobox 2	myogenesis regulation	4223
*GABRE*	Gamma-aminobutyric acid (GABA) A receptor, epsilon	synaptic transmission	2564
*CEACAM1*	Carcinoembryonic antigen-related cell adhesion molecule 1 (biliary glycoprotein)	cell adhesion	634
*BET1*	Bet1 golgi vesicular membrane trafficking protein	vesicular transport	10282
*MXRA5*	Matrix-remodelling associated 5	matrix remodelling	25878
*IGFBP7*	Insulin-like growth factor binding protein 7	cell growth and differentiation	3490
*NETO2*	Neuropilin (NRP) and tolloid (TLL)-like 2	cell signaling	81831
*BAI3*	Brain-specific angiogenesis inhibitor 3	angiogenesis	577
*PLXDC1*	Plexin domain containing 1	uncharacterized	57125
*JAG1*	Jagged 1	hematopoiesis	182
*EDNRA*	Endothelin receptor type A	cell signaling	1909
*ICAM2*	Intercellular adhesion molecule 2	cell adhesion	3384
*NOTCH4*	Notch 4	cell fate	4855
*STAB1*	Stabilin 1	cell growth and differentiation	23166
*EPHB3*	EPH receptor B3	cell signaling	2049
*LPHN1*	Latrophilin 1	cell adhesion/signal transduction	22859
*NPR1*	Natriuretic peptide receptor 1	cell signaling	4881
Under-expressed			
*GPR37*	G protein-coupled receptor 37 (endothelin receptor type B-like)	cell signaling	2861
*IGFBP3*	Insulin-like growth factor binding protein 3	cell growth and differentiation	3486
*FLT1*	Fms-related tyrosine kinase 1	cell growth and differentiation	2321
*PDGFRA*	Platelet-derived growth factor receptor, alpha polypeptide	cell growth and differentiation	5156
*TGFBR3*	Transforming growth factor, beta receptor III	cell growth and differentiation	7049
*LPAR1*	Lysophosphatidic acid receptor 1	cell growth and differentiation	1902
*IGFBP5*	Insulin-like growth factor binding protein 5	cell growth and differentiation	3488
*EDNRB*	Endothelin receptor type B	cell signaling	1910
*PDGFC*	Platelet derived growth factor C	cell growth and differentiation	56034
*BMP4*	Bone morphogenetic protein 4	cell growth and differentiation	652
*ANGPTL1*	Angiopoietin-like 1	cell growth and differentiation	9068
*VCAM1*	Vascular cell adhesion molecule 1	cell adhesion	7412
*BMP5*	bone morphogenetic protein 5	cell growth and differentiation	653
*IGF1R*	Insulin-like growth factor 1 receptor	cell growth and differentiation	3480
*ANGPT2*	Angiopoietin 2	vascular remodeling	285
*ANTXR1*	Anthrax toxin receptor 1	cell signaling	84168
*CLDN11*	Claudin 11	cell adhesion	5010
*KISS1*	KiSS-1 metastasis-suppressor	cell adhesion	3814

**Fig. 2:**
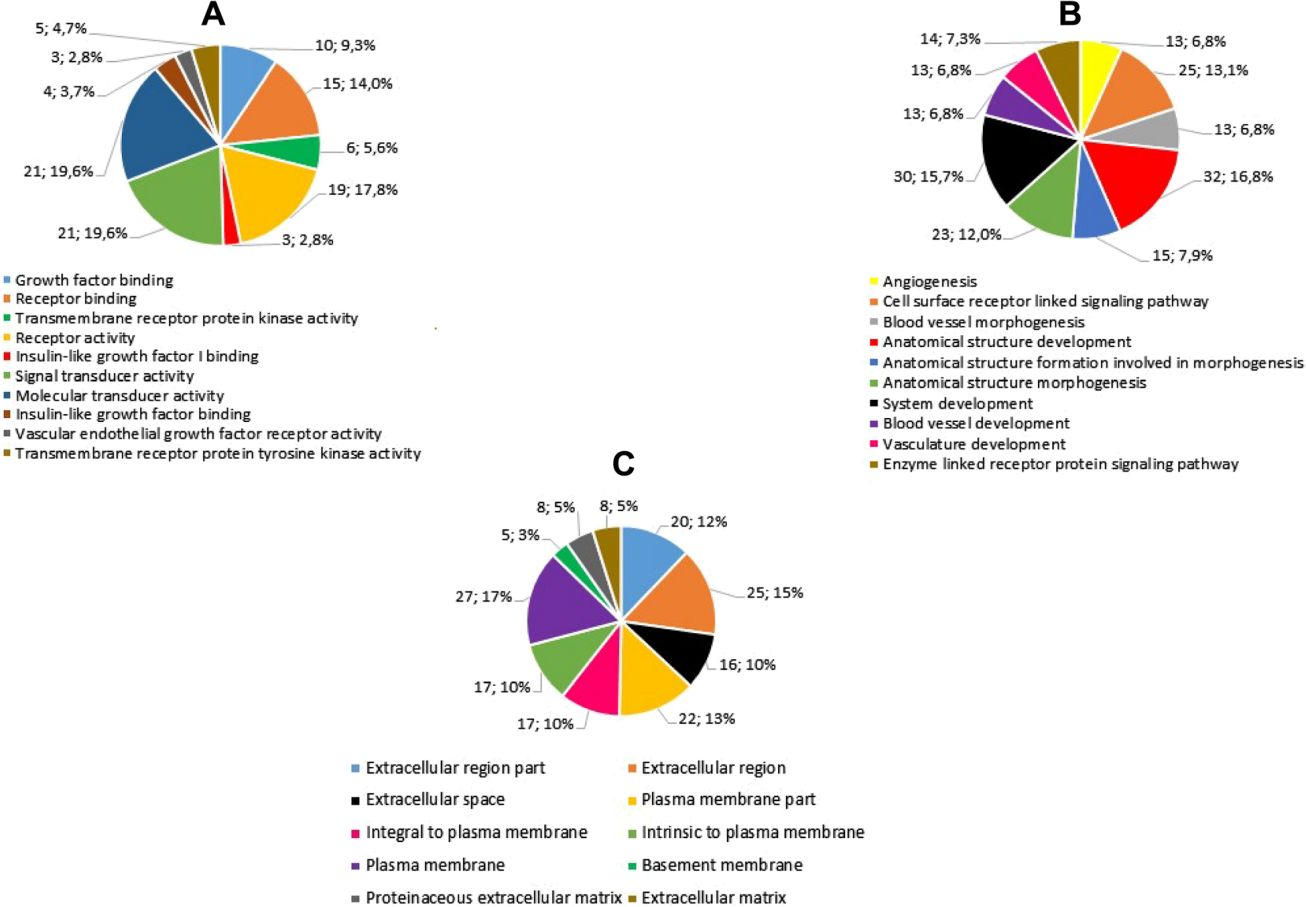
The top 10 enriched Gene Ontology terms of differentially expressed genes. **a**. molecular function (P value ≤ 1,87E-02); **b**. biological process (P value ≤ 3,98E-08); **c**. cellular component (P value ≤ 7,98E-02)

**Fig. 3:**
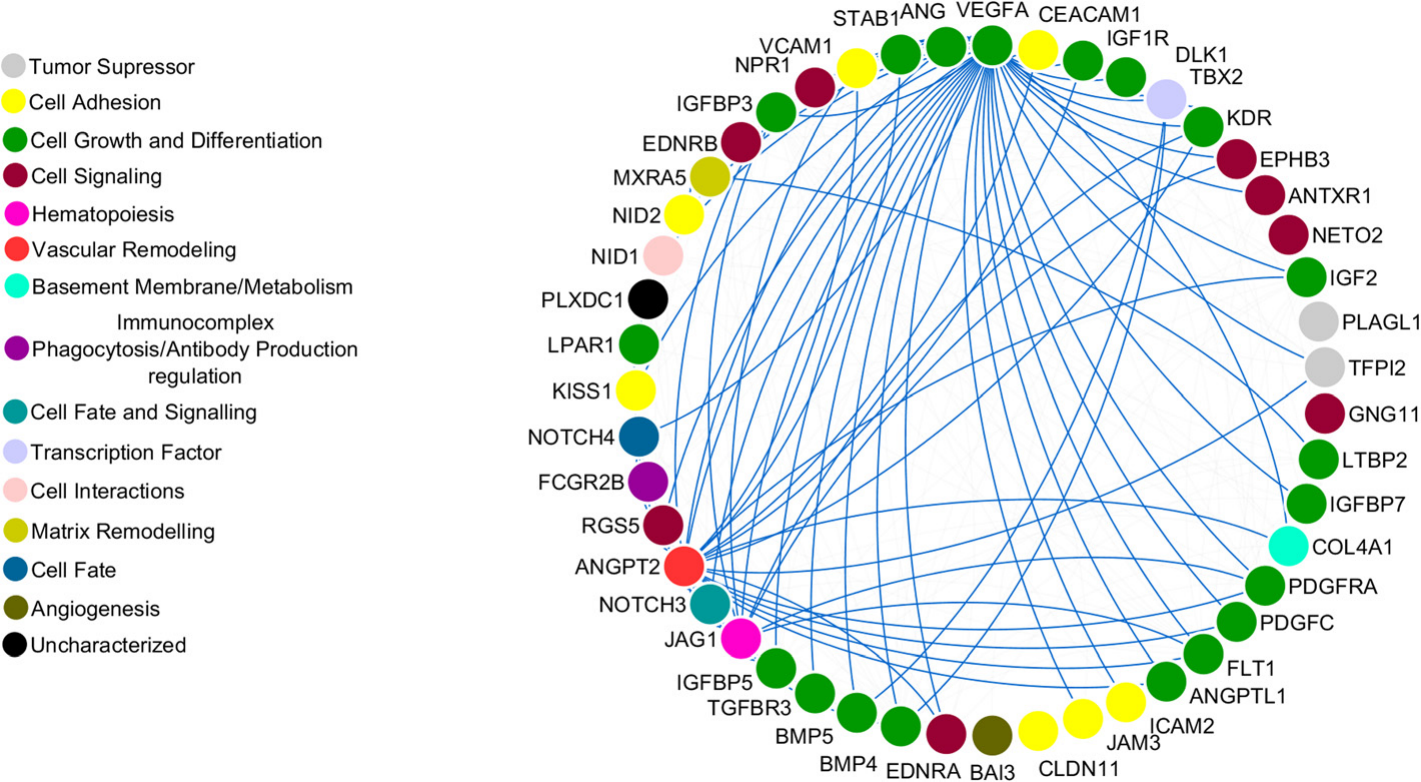
Protein-protein interaction network. The blue lines highlight the interactions of VEGFA, TBX2, JAG1, ANGPT2 and MXRA5. STRING v9.1 was used to generate protein interactions and the result of the network was visualized using Cytoscape v3.1.1

**Fig. 4:**
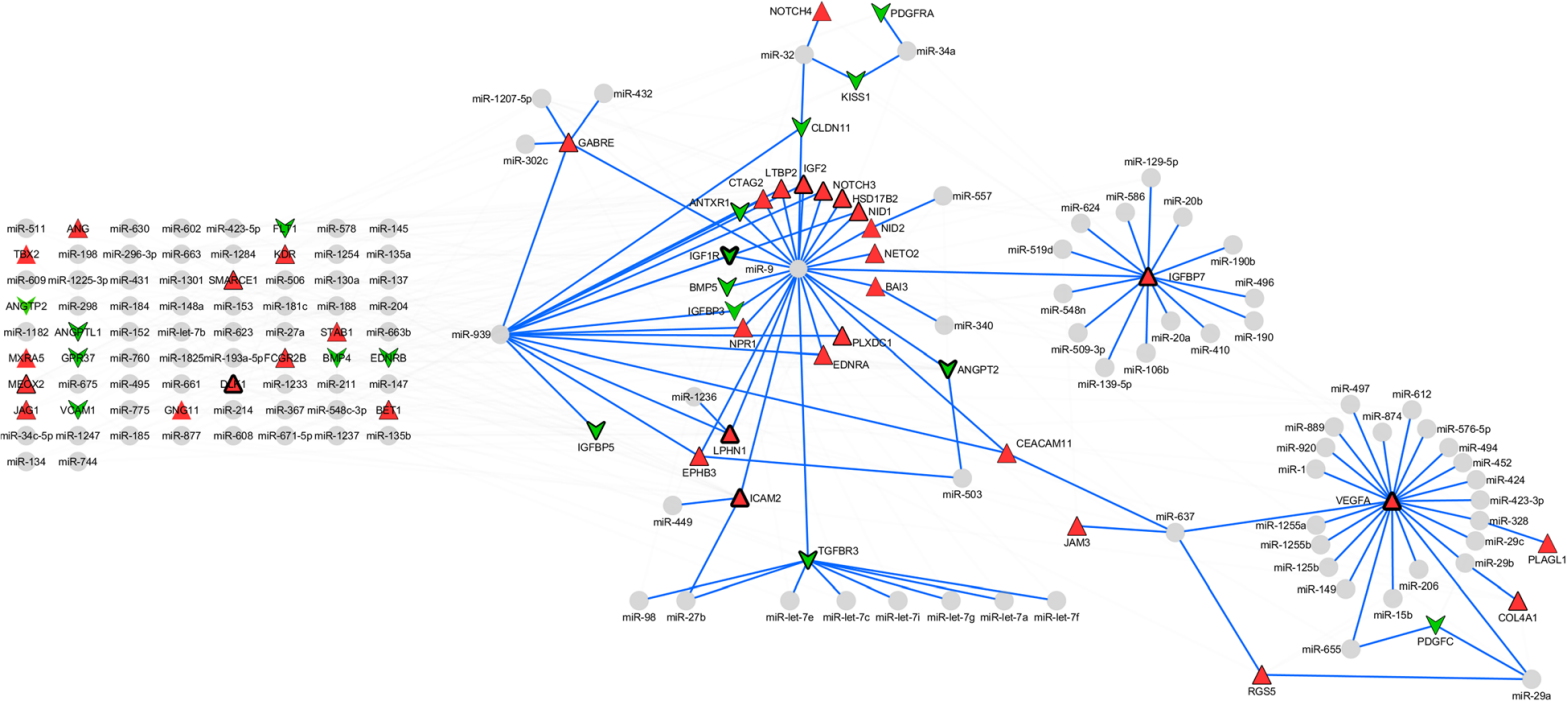
miRNA-gene interaction network. The larger the triangle edge, the higher is the number of interactions identified. The gray circles are miRNAs and blue lines highlights the interactions. The grid only groups miRNAs and genes with few interactions. The miRNA-gene interactions were visualized using Cytoscape v3.1.1

Enrichment pathways analysis showed information on the biological role of differentially expressed genes in infantile hemangioma. Gene Ontology (GO) categories were divided into 3 hierarchically structured groups, in order to identify proteins encoded by deregulated genes in infantile hemangioma, and associated with biological processes, molecular functions and cellular components. The top 10 statistically significant enriched GO terms are shown in Fig. 2. Integrated, complex interactome analysis for deregulated genes in infantile hemangioma and functional annotations are shown in Fig. 3. A higher number of interactions was identified between genes with roles in vascular and matrix remodeling, hematopoiesis, cell growth and differentiation and transcriptional control. An interaction network between genes and miRNAs predicted to regulate the expression of these specific genes is shown in Fig. 4. The red and green triangles represent up-regulated and down-regulated genes, respectively. Notably, DGIdb data showed that 5 genes were predicted to interact with drugs that have been demonstrated as clinically useful in other tumor types (Table 3).

**Table 3:** Potential target agents identified based on protein-protein interaction networks of deregulated genes in infantile hemangioma

Gene symbol	Gene name	Selected target agent	Clinical relevance
*EDNRA*	endothelin receptor type A	Zibotentan, Atrasentan	Colorectal [62], prostate [63] and renal cell [64] carcinomas
*IGF1R*	insulin-like growth factor 1 receptor	Linsitinib, Ganitumab, Figitumumab, Dalotuzumab, Cixutumumab, Robatumumab	Adrenocortical [65], ovarian [66], non-small cell lung [67], colorectal [68, 69] carcinomas and soft tissue sarcoma [70]
*PDGFC*	platelet derived growth factor C	Sunitinib	Renal cell carcinoma [71] and breast carcinomas [72]
*PDGFRA*	platelet-derived growth factor receptor, alpha polypeptide	Motesanib, Ramucirumab, Midostaurin, Amuvatinib, Nintedanib, Pazopanib, Tandutinib, Crenolanib, Nilotinib, Masitinib, Sorafenib, Sunitinib, Regorafenib, Dovitinib, Telatinib, Vatalanib, Axitinib, Lenvatinib, Imatinib	Colorectal [73, 74], hepatocellular [75–78], kidney [79], non-small [80] and small cell lung [81], pancreatic [82–84], colon [85], gastrointestinal [86], renal cell [71, 87], breast [72, 88], melanoma [89], thyroid [90] carcinomas and soft tissue sarcoma [91]
*VEGFA*	vascular endothelial growth factor A	Ziv-aflibercept, Bevacizumab, Sorafenib tosylate, Lenalidomide, Thalidomide, Aflibercept	Colorectal [92, 93] ovarian [94], non-small cell lung [95], hepatocellular [96] carcinomas and multiple myeloma [97]

## Discussion

### Molecular pathways deregulated in hemangioma

Molecular pathogenesis of infantile hemangioma is not well understood. Advances in methods of global genetic and epigenetic analyses represent an extremely valuable approach for the identification of disease development mechanisms and have the potential to identify biomarkers and/or pathways that may be useful for the development of better treatment approaches, including molecularly-targeted therapies.

Our meta-analysis approach allowed us to integrate mRNA expression data in infantile hemangioma and to predict which miRNAs are potential regulators of gene expression. Among the different mechanisms that can lead to gene expression alterations; miRNA alteration is an important mechanism of over- or under-expression of target genes [48]. Herein, we aimed to utilize data on deregulated genes in infantile hemangioma, in order to predict which miRNAs could potentially regulate these genes, and to construct interaction networks between genes and miRNAs.

Gene enrichment analysis showed that deregulated genes previously reported in infantile hemangioma [31–37] are mainly involved in cell signaling and angiogenesis, functioning in vascular and matrix remodeling, hematopoiesis, cell growth and differentiation and transcriptional control.

It is known that the formation of vascular tumors including infantile hemangioma is partly related to increased expression of angiogenic growth factors, such as basic fibroblast growth factor (bFGF) and vascular endothelial growth factor (VEGF), which lead to the development of a disorganized blood vessel mass [49]. Indeed, angiogenesis is mainly regulated by the vascular endothelium [50].

miRNAs control and modulate cell response of vascular endothelium to angiogenic stimuli; for example, miR-126 is a positive regulator of angiogenic signaling and vascular endothelial integrity. Vascular development defects were demonstrated in an in vitro model of miR-126-depleted cells, which did not respond to bFGF and VEGF angiogenic factors [51]. Angiogenic response is also controlled by miRNAs, such as miR-221 and miR-222, which play a role as inhibitors of stem cell factors. Other miRNAs, such as miR-27b and miR-let-7f, play a pro-angiogenic role, since their expression promotes angiogenesis [51]. Notably, miRNA expression in vascular endothelial cells can be modified in response to cellular stimuli or to the microenvironment. For example, a hypoxic environment promotes the production of miR-210, which has pro-angiogenic activity. Therefore, increase in pro-angiogenic miRNA expression in endothelial cells may stimulate the production of angiogenic factors, contributing to the process of tumorigenesis [51].

VEGFA plays an important role in vascular development and in pathological angiogenesis and its protein is highly expressed in vessels of proliferating infantile hemangioma [52]. Interestingly, angiogenin protein (ANG), which is required for cell proliferation and is an important mediator of blood vessel formation, regulates VEGFA expression [53]. Although VEGFA was not identified among the deregulated genes reported in the studies used for meta-analysis, VEGFA is shown in the PPI and miRNA-gene interaction networks, likely due to its important role in angiogenesis and to its indirect interaction with other proteins in the network.

To our knowledge, the only available previously published study on miRNAs in hemangioma identified miR-424 under-expression in senile hemangioma [39]. miR-424 is shown interacting with VEGFA in our miRNA-gene network analysis. miR-424 over-expression has been associated with greater cell motility, decreased cell adhesion and other alterations associated with epithelial-to-mesenchymal transition (EMT) [54]. Notably, this study showed that miR-424 expression levels are increased in primary tumors and decreased in metastasis compared to primary breast tumors and additional functional data suggested that miR-424 may play different roles in the different stages of tumor development and progression [54].

Several genes with roles in vascular and matrix remodeling, hematopoiesis, cell growth and differentiation and transcriptional regulation were shown in the PPI and miRNA-gene interation network. Among these, ANGPT2, MXRA5, JAG1, VEGFA and TBX2 showed a large number of interactions. Interestingly, some of these genes also play roles in cell signaling pathways that have been linked to the pathogenesis of infantile hemangioma [55]; namely, VEGFA in the VEGF/VEGFR pathway, ANGPT2 and ANGPTL1 in the Tie2/Angiopoietin signaling pathway and NOTCH3, NOTCH4 and JAG1 in the Notch pathway. Growth factors and angiopoietins have roles in embryonic development and angiogenesis-dependent diseases and Notch components are involved in modulation of cell fate and differentiation [55].

Herein, miRNA-gene interaction networks generated by integrative meta-analysis showed miRNAs with a large number of interactions (miR-9, miR-939, and let-7 family of miRNAs); these miRNAs are likely acting as main regulators in the network. Notably, miR-9 has been demonstrated as pro-metastatic and suppressor of E-cadherin in breast cancer cells, promoting cell motility and increasing invasive potential of carcinoma cells, besides activating β-catenin signaling, which in turn contributes to high VEGFA expression and consequently to induction of angiogenesis [56]. Increased miR-9 expression levels were also associated with EMT in breast cancer cells and with poor prognosis of patients with breast cancer [57]. In ovarian cancer, miR-939 plays an important role in the progression and regulation of cell growth and cell cycle; it has been demonstrated that ES-2 cells transfected with miR-939 mimic show APC2 decreased expression, suggesting that APC2 may be a target of miR-939 [58].

A recent meta-analysis suggested that the let-7 family of miRNAs are potential biomarkers for tumor grade prediction in breast cancer [59] as well as in other cancers, since let-7 family is highly conserved across species [60].

Among other miRNAs with a significant number of interactions, miR-637 links to main networks through direct interactions with VEGFA and CEACAM; the latter interacting with miR-9 and miR-939. Functional data has shown that miR-637 is one of the effective regulators of HER2 signaling; in HER2-positive trastuzumab non-responsive cell lines, miR-637 was efficient to inhibit breast cancer cell growth [61].

## Conclusion

Herein, we identified several interconnected genes and miRNAs as potential regulators of gene expression. Such miRNAs and genes may play important roles in the development and progression of infantile hemangioma. Additionally, these molecules show potential to be targets for drugs that may be clinically useful in the development of new therapies for infants and children affected by this tumor. Data generated herein may be used for validation of expression of miRNAs and genes regulated by miRNAs in infantile hemangioma. Validation analysis in a large representative cohort of primary untreated patient samples is necessary in order to establish robust biomarkers for prediction of treatment response and for the development of better treatment modalities.

## Abbreviations

miRNA or miR: microRNA
miRDIP: microRNA data integration portal
BiNGO: biological networks gene ontology
GO: gene ontology
PPI: protein-protein interaction
DGIdb: drug-gene interaction database
TTD: therapeutic target database
PharmaGKB: pharmacogenomics knowledge base
bFGF: basic fibroblast growth factor
VEGF: vascular endothelial growth factor
ANG: angiogenin protein
EMT: epithelial-to-mesenchymal transition
QRT-PCR: quantitative reverse-transcription polymerase chain reaction
HEMECs: Infantile hemangioma endothelial cells
HDMVECs: dermal microvascular endothelial cells
HemECs: hemangioma-derived endothelial cells
hemSCs: proliferating hemangioma-derived CD133+ cells
HDMECs: human dermal microvascular endothelial cells
cbEPCs: cord blood endothelial progenitor cells
abEPCs: adult blood endothelial progenitor cells
bm-MPCs: bone marrow-mesenchymal progenitor cells
HemSCs: hemangioma-derived stem cells
MSCs: mesenchymal stem cells
